# The GATK joint genotyping workflow is appropriate for calling variants in RNA-seq experiments

**DOI:** 10.1186/s40104-019-0359-0

**Published:** 2019-06-21

**Authors:** Jean-Simon Brouard, Flavio Schenkel, Andrew Marete, Nathalie Bissonnette

**Affiliations:** 1 Sherbrooke Research and Development Centre, Agriculture and Agri-Food Canada, Sherbrooke, QC J1M 0C8 Canada; 2 Center of Genetic Improvement of Livestock, University of Guelph, Guelph, ON N1G 2W1 Canada

**Keywords:** GATK, GVCF, Joint genotyping, RNA-seq, SNP

## Abstract

The Genome Analysis Toolkit (GATK) is a popular set of programs for discovering and genotyping variants from next-generation sequencing data. The current GATK recommendation for RNA sequencing (RNA-seq) is to perform variant calling from individual samples, with the drawback that only variable positions are reported. Versions 3.0 and above of GATK offer the possibility of calling DNA variants on cohorts of samples using the HaplotypeCaller algorithm in Genomic Variant Call Format (GVCF) mode. Using this approach, variants are called individually on each sample, generating one GVCF file per sample that lists genotype likelihoods and their genome annotations. In a second step, variants are called from the GVCF files through a joint genotyping analysis. This strategy is more flexible and reduces computational challenges in comparison to the traditional joint discovery workflow. Using a GVCF workflow for mining SNP in RNA-seq data provides substantial advantages, including reporting homozygous genotypes for the reference allele as well as missing data. Taking advantage of RNA-seq data derived from primary macrophages isolated from 50 cows, the GATK joint genotyping method for calling variants on RNA-seq data was validated by comparing this approach to a so-called “per-sample” method. In addition, pair-wise comparisons of the two methods were performed to evaluate their respective sensitivity, precision and accuracy using DNA genotypes from a companion study including the same 50 cows genotyped using either genotyping-by-sequencing or with the Bovine SNP50 Beadchip (imputed to the Bovine high density). Results indicate that both approaches are very close in their capacity of detecting reference variants and that the joint genotyping method is more sensitive than the per-sample method. Given that the joint genotyping method is more flexible and technically easier, we recommend this approach for variant calling in RNA-seq experiments.

**Electronic supplementary material:**

The online version of this article (10.1186/s40104-019-0359-0) contains supplementary material, which is available to authorized users.

## Main text

Mainly designed to quantify gene expression, the next-generation sequencing (NGS) of RNA samples (RNA sequencing, or RNA-seq) also offers new opportunities for the efficient detection of transcriptome variants (SNPs and short indels). RNA-Seq notably represents a powerful approach for discovering causal mutations underlying quantitative trait loci [1]. Recent examples include the transcriptome analysis of the bovine pituitary gland [2], bovine blastocysts [3], pig hypothalamus and liver [4]. RNA-seq can also generate a large number of genotypes required to test the association of polymorphisms with traits of economic importance [5]. However, several precautions must be taken when calling variants from RNA-seq data. The main challenges include handling splice junctions, detecting variants in low-expressed regions, and managing duplicated reads [6, 7]. Many of the numerous strategies and tools proposed to overcome these challenges rely on the Genome Analysis Toolkit (GATK), which is a popular set of programs for discovering and genotyping variants from next-generation sequencing (NGS) data [8, 9]. The group behind GATK published the GATK Best Practices for variant calling, which are essentially a number of optional steps that were proven to increase the quality of NGS-derived variants, steps either upstream (preparatory) or downstream (filtering) of the variant calling process [10]. The current GATK recommendation for RNA-seq data is to perform variant calling from individual samples [11]. This approach has the drawback that only variable positions are reported in variant calling format (VCF) files, because otherwise too many positions would be reported. Thus, homozygous genotypes for the reference allele are not called and cannot be distinguished from missing data, a major issue in the preparation of datasets for genome-wide association study applications. Versions 3.0 and above of GATK offer the possibility of calling germline variants on cohorts of samples using the HaplotypeCaller algorithm in GVCF mode [12]. This strategy is more flexible and reduces computational time in comparison with the traditional joint discovery workflow, especially when large and growing cohorts of samples are being worked with. Using a joint genotyping workflow with RNA-seq can provide substantial advantages over the individual calling method, including reporting all genotype types as well as missing data in a single VCF file. The joint genotyping workflow consists of processing RNA-seq samples in accordance with the GATK Best Practices workflow for variant calling on RNA-seq data up to the variant calling step and then switching to the joint variant workflow in the HaplotypeCaller stage; this approach will be referred as the “joint genotyping method” thereafter. The joint genotyping method was validated using a pairwise comparison approach by evaluating its sensitivity, precision, and accuracy in genotype calling. The per-sample method was basically the GATK individual calling method for RNA-seq data plus improvements to add homozygote calls retrieved using a mpileup + BCFtools call pipeline (Additional file 1: Figure S1).

**Fig. 1:**
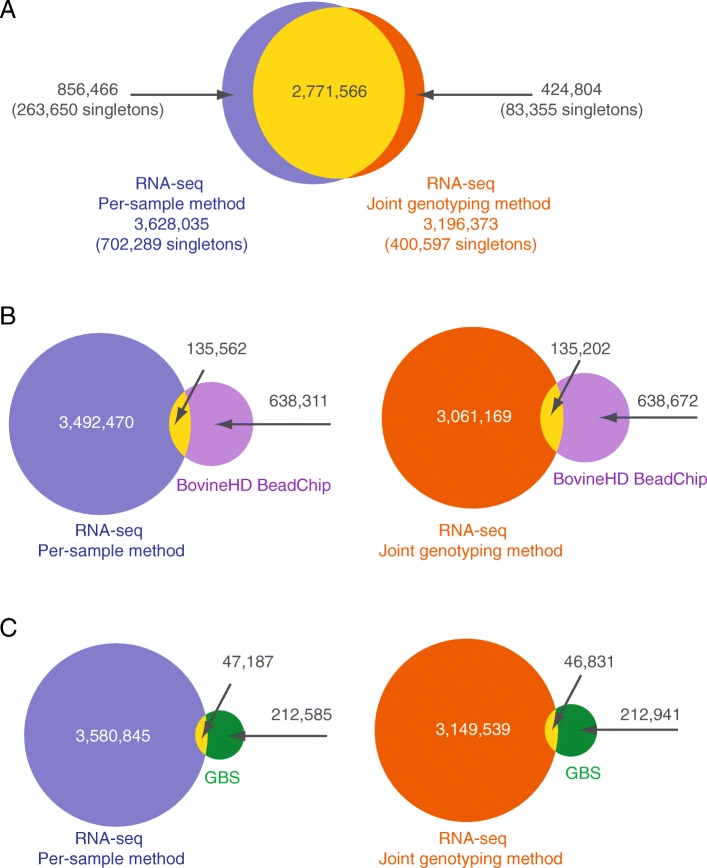
Common variants found in different datasets. **a** Comparison of RNA-seq variants detected using the per-sample and the joint genotyping approaches. **b** Comparison of the two sets of RNA-seq variants with those detected by the BovineHD BeadChip. **c** Comparison of the two sets of RNA-seq variants with those detected by GBS

The RNA-seq data from the 50 cows analyzed in this study yielded 3,628,035 unique variants for the per-sample method and 3,196,373 for the joint genotyping method, while 2,771,566 variants were detected by both methods (Fig. 1a). This result is not particularly surprising since it is well known that different SNP calling algorithms always find unique sets of variants [13, 14]. In addition, one should keep in mind that the number of variants reported in Fig. 1a refer to variants that were not validated. One can suspect that many of these variants are false positives. Notwithstanding, to tentatively explain why the number of variants reported by the joint-genotyping approach is lower we examine the hypothesis that the joint-genotyping approach can miss a small fractions of singletons, i.e. variants unique to individuals samples [15, 16]. We tested this hypothesis by simply counting the number of singletons present in each datasets. Results indicate that the joint-genotyping actually detect less singletons than the per-sample method (400,597 vs 702,289). In variants private to the per-sample method, the proportion of singletons reach 32% (263,650/856,466), more than the 19% (83,355/424,804) found in variants private to the joint-genotyping method. This factor contribute to the lower number of variants reported by the joint-genotyping. However in many applications like GWAS, singletons are not much important and are likely to be filtered out owing to their very low call rate values. We also performed pairwise comparisons of the two sets of RNA-seq variants to sets of variants identified from the same 50 cows using two other sources: those genotyped using the BovineSNP50 Beadchip and imputed to the BovineHD Beadchip (Fig. 1b), and those identified through a previously described two-enzyme genotyping-by-sequencing (GBS) assay (Fig. 1c) [17]. Of the 777,962 markers present on the BovineHD array, 135,562 were identified by the per-sample method, and 135,201 were identified by the joint genotyping method (Fig. 1b) in conditions were genotypes call with less than 5 reads were removed. GBS-derived variants were also found in the two RNA-seq datasets: 47,187 variants were common with the per-sample method, and 46,831 were also detected using the joint genotyping method (Fig. 1c). Together, these results clearly illustrate that both approaches are very close in their capacity of detecting reference variants, either BovineHD or GBS variants.

**Fig. 2:**
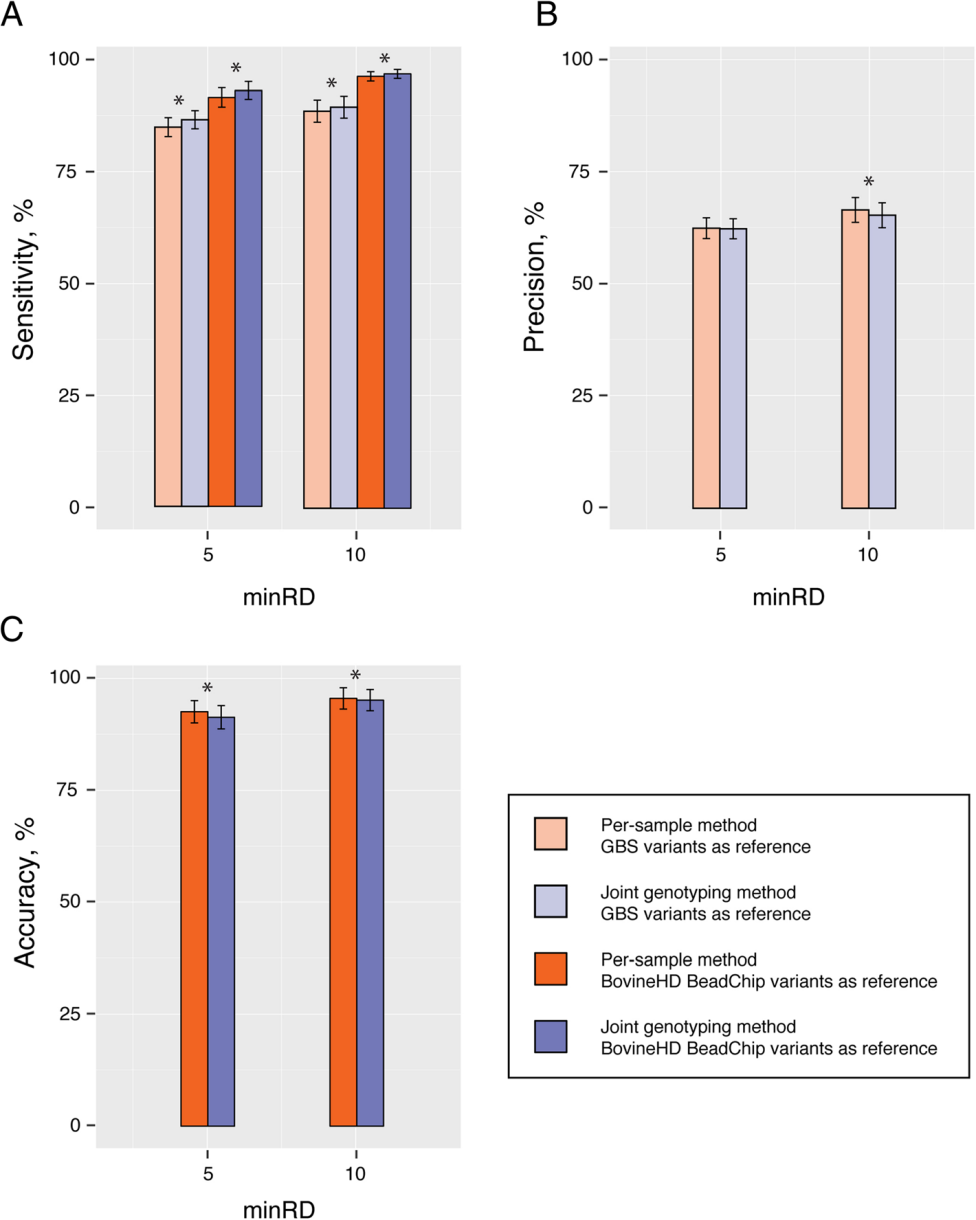
Effect of the minimum read depth (minRD) on the (**a**) sensitivity, (**b**) precision, and (**c**) accuracy of genotype calls of two RNA sequencing (RNA-seq) calling approaches. While the precision is a measure of true positives, accuracy is considered as a false negative measurement. The analysis was performed at different minRD on each sample. Error bars represent two times the individual standard error at each minRD. Asterisks indicate a significant effect at *P <* 0.05 using a Wilcoxon nonparametric test. The minRD is the minimum number of reads that need to be present in an RNA-seq or genotyping-by-sequencing (GBS) region in order for the region to be considered in the analysis

Because we genotyped the same 50 animals with ‘three’ methods, we have a unique opportunity to validate the variants and the genotypes detected by RNA-SEQ. DNA variants obtained by GBS and those from the BovineHD Beadchip were used as reference for evaluating the sensitivity, the precision, and the accuracy of genotype calls of the two variant calling approaches. The calculation of the sensitivity, precision and accuracy of genotypes was performed for each variant calling method, using one sample at a time and at minimum read depth (minRD) coverage of 5 and 10. The definition of the parameters (sensitivity, precision and accuracy) and scripts can be found in Additional file 2 and Additional file 3, respectively. Results indicate that at relatively high read depth coverages (minRD = 5, or 10), the joint genotyping method had a slightly but significantly better capacity to detect variations than the per-sample method had, although the genotypes produced with the joint genotyping method were less accurate (Fig. 2c; *P <* 0.05).

The sensitivity was consistently lower when the Genotyping-by-Sequencing (GBS) variants were used as references (Fig. 2a). BovineHD variants are considered to be the “gold standard” for assessing the sensitivity and accuracy of genotype calls [13]. In this study, imputed BovineHD genotypes were used instead. However, the imputation was expected to be highly accurate, with both average concordance rate and allelic r^2^ higher than 0.99 [18]. In contrast, the GBS-derived variants were assumed to be more suitable for assessing precision, which is a measure of false positives, because GBS variants make it possible to test not ascertained sites for the presence of variations.

We found that the most striking differences between the two methods were observed in conditions where very low-coverage regions (minRD < 5) were included in the analysis (data not shown), a situation corresponding to the default output of the GATK workflows. However, regions supported by only one or two reads should be considered with caution for variant calling. Indeed, low-coverage sequencing introduces uncertainty into the results and makes SNP detection and genotype calling difficult [19, 20]. Our analyses indicate that gains in sensitivity, in the precision of variant calling, and in the accuracy of genotype calls can be obtained by slightly increasing the minimal threshold of reads required for variant calling (Fig. 2).

On the other hand, being too stringent about the minimal number of reads required for variant calling would be counterproductive, since too many variants would be filtered out. Notwithstanding, a greater precision can be reached using the Per-sample method at minRD = 10 (Fig. 2b). There is no conclusive explanation, but it can be speculated that the difference observed at high expression levels could have been induced by a differential allelic expression in some individuals that was missed with the joint genotyping method missed. Although GBS does not account for the abundance of the genotyped alleles, the RNA-seq reads reflect this abundance. Further investigation will be needed to clarify this matter, which was not the goal of this communication. The tradeoff between the number of variants retained after filtration (e.g. minRD = 5) and the variant quality has been observed in GBS [17, 21] and in many NGS applications that rely on the principle that sequencing a large number of individuals at low-coverage depths is a better approach than sequencing fewer individuals at high-coverage depths [22].

Analyzing samples together is almost always considered a better strategy than analyzing them individually, because the former method is expected to take advantage of population-wide information and lead to improved sensitivity in variant detection and a higher accuracy of genotype calls [10]. The sensitivity measurements reported here (Fig. 2a at minRD = 5, or 10) are consistent with this idea and are in line with previous studies that have shown a slight improvement in sensitivity (1% to 4%) when variant calling was performed using multi-sample methods rather than single-sample methods [23].

## Conclusion

In summary, the GATK joint genotyping approach with RNA-seq data was validated using a large number of samples genotyped with alternative techniques. The joint genotyping method can be used with confidence in most contexts, since researchers will generally want to exclude poor-quality genotypes called with only one or two reads and not restricting SNP calling to only highly expressed SNP (minRD ≥10). In these conditions, the joint genotyping method has a greater capacity to call with good sensitivity a substantially higher number of variants than the per-sample method. The tradeoff is to have lower accuracy but higher sensitivity using an approach that is technically simpler and much less computationally demanding. Furthermore, as shown in [14], there is a tradeoff between accuracy and objectives of downstream analysis. Should the objective be GWAS analysis, then combining several variant callers and taking advantage of the long-range linkage disequilibrium in dairy cattle to impute the missing genotypes has been reported as a viable option [24, 25].

## Additional files

Additional file 1: **Figure S1.** Schematic representation of the method used for adding homozygote calls (0/0) corresponding to the reference allele to the RNA-seq per-sample dataset. (PDF 159 kb)

Additional file 2: Materials and Methods. (DOCX 24 kb)

Additional file 3: Bioinformatics scripts used in this study. (DOCX 18 kb)

## Abbreviations

GATK: Genome Analysis Toolkit
GBS: Genotyping-by-sequencing
GVCF: Genomic variant call format
minRD: Minimum read depth
NGS: Next-generation sequencing
RNA-seq: Ribonucleic acid sequencing
SNPs: Single nucleotide polymorphisms
