# Supplementary figures and images for: The GATK joint genotyping workflow is appropriate for calling variants in RNA-seq experiments
Source: J Anim Sci Biotechnol. 2019 Jun 21;10:44. doi: 10.1186/s40104-019-0359-0 (PMC6587293; doi:10.1186/s40104-019-0359-0)

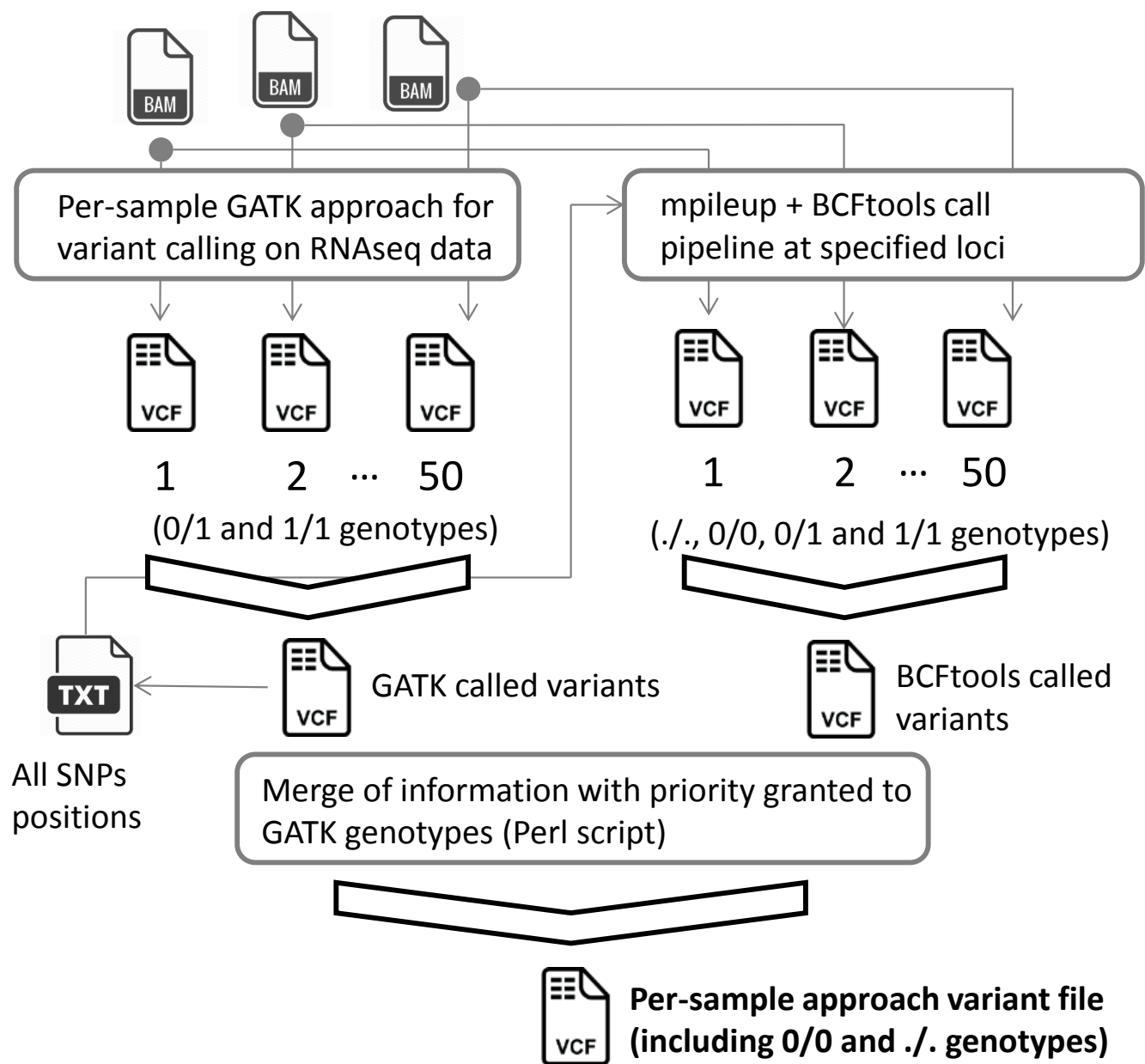

**Fig. S1**

Supplement: Supplementary file 1 — Figure S1. Schematic representation of the method used for adding homozygote calls (0/0) corresponding to the reference allele to the RNA-seq per-sample dataset. (PDF 159 kb) [file 40104_2019_359_MOESM1_ESM.pdf]
