# Supplementary material for: The GATK joint genotyping workflow is appropriate for calling variants in RNA-seq experiments
Source: J Anim Sci Biotechnol. 2019 Jun 21;10:44. doi: 10.1186/s40104-019-0359-0 (PMC6587293; doi:10.1186/s40104-019-0359-0)
Supplement: Supplementary file 2 — Materials and Methods. (DOCX 24 kb) [file 40104_2019_359_MOESM2_ESM.docx]

# MATERIAL AND METHODS

## RNA-seq data processing

RNA was extracted from whole primary macrophages from a total of 50 cows. Among these 50 cows, 11 cows were selected for studying gene expression in the context of Bovine Paratuberculosis. For each of the 11 cows, sequence data from 6 distinct libraries were pooled to produce FASTQ files containing roughly 360 million paired-end (PE) reads per cow. Otherwise, sequence files from the 39 additional cows contained a minimum of 60 million PE reads. An average of 181 million of unique paired-end reads per sample were mapped against the bovine reference genome assembly (UMD_3.1.1/BosTau8) using the STAR aligner (version 2.4.0j; [1] ). Next, duplicated reads were marked with Picard tools. The recommended Split’N’Trim and indel realignments steps were also performed (GATK, version 3.3-0-g37228af).

## SNP calling – Per sample method

SNPs and indels were first identified from RNA-seq data by a single sample method using the Genome Analysis Toolkit (GATK, [2] ) in accordance with the Best Practices workflow for variant calling on RNA-seq data, except that variant filtering was not performed at this step. Notice that the output VCF files produced at this step completely lack homozygote genotypes for the reference allele. However, there was interest in adding this information to the final dataset and clearly distinguish it from missing data. In this context, missing data would represent genotypes that are unknown because their corresponding loci are not covered by any read in a specific alignment file. This procedure is summarized in Fig. S1. Briefly, all VCF files produced by the GATK single sample method were merged and reported all SNP positions in a text file. Using a mpileup + BCFtools call pipeline restricted to SNP regions listed in this file, a second calling procedure was performed to produce VCF files with all types of genotype information, including homozygote genotypes for the reference allele as well as missing data. Using a custom Perl script, all genotypes (mostly homozygotes calls for the reference allele) that were absent in the GATK merged VCF file but present in the merged BCFtools VCF file were added to the GATK merged VCF file. The final dataset was filtered out to replace genotypes called with less than 5 reads by missing data.

## SNP calling – Joint genotyping method

Using the same alignment files that were used for the Per sample method, the steps of the joint variant discovery workflow as described in the Best practices for Germline SNP & Indel discovery in Whole Genome and Exome sequence [3] were followed. With this 2-step approach, firstly, potential variants were called on each sample using the –ERC GVCF mode, leading to the production of one gVCF file per sample that lists genotype likelihoods and genome annotations. Secondly, variants were called through a Joint Genotyping analysis from all gVCF files. As recommend by the GATK Best Practices, we used the HaplotypeCaller algorithm to call variants. The following command was run for each sample: java –Xmx16g –jar GenomeAnalysisTK.jar –T HaplotypeCaller –R UMD3.1.fa –I sample.bam –nct 4 –variant_index_type LINEAR – variant_index_parameter 128000 –emitRefConfidence GVCF –o raw.snps.indels.g.vcf. With all the GVCFs files derived from the above mentioned step, a Joint Genotyping analysis was performed for all samples and one final VCF file was produced. The following command was used: java –Xmx256g –jar GenomeAnalysisTK.jar –T GenotypeGVCFs –R UMD3.1.fa --variant sample1.raw.snps.indels.g.vcf -- variant sample2.raw.snps.indels.g.vcf etc. ~~Again, raw variants were used for the analysis, as variant filtering was not performed at this step.~~ Again, the final dataset was filtered out to replace genotypes called with less than 5 reads by missing data.

## DNA genotyping using Bovine SNP50 BeadChip and Genotyping-by-sequencing

All 50 cows analyzed in RNA-seq were also genotyped with the commercial Illumina BovineSNP50 BeadChip (Zoetis, Kalamazoo, MI) and using the Genotyping-By-Sequencing technique as already described [4]. Data from the SNP50 Beadchip were imputed to the BovineHD BeadChip density using FImpute [5] and a large reference population of 3,011 animals. GBS sequence data (100-nt fastq files) were processed using the Fast-GBS pipeline [6] with the GATK HaplotypeCaller using the defaults parameters.

## Methodology to evaluate the precision, the sensitivity and the accuracy of genotype calls

Taking advantage of the availability of alternative genotyping data for all 50 samples analyzed in RNA-seq, i.e. variants detected in GBS and those present on the BovineHD BeadChip as reference variants, the sensitivity, precision, accuracy of genotype calls of both variant calling method were compared. For each sample, only assembled regions covered by a minimal number of reads in RNA-seq (when working with BovineHD variants as reference) or regions covered by a minimal number of reads in RNA-seq and GBS (when working with GBS variants as reference) were kept for the analysis. Sequence depth coverage was firstly evaluated by the BEDtools genomecov command then regions covered with a minimum number of reads (minRD) were identified using a custom Perl script (Additional file 2).

Sensitivity, precision, and accuracy of both SNP variant calling methods were evaluated based on the RNA-seq and DNA common variants detected within at least 10 individuals (call rate ≥ 0.2). DNA variants were obtained using the BovineHD beadChip genotypes or the GBS variants as reference. To assess the genotype calls from many samples generated to represent different minimal read depth coverage a bash pipeline was developed, which is available in Appendix S2. Throughout this pipeline, variants were compared using the BCFtools isec command.

The sensitivity was evaluated using either the BovineHD beadChip variants or the GBS variants as reference and the following equation:

Sensitivity = 100*TP/(TP+FN)

Where TP = Reference variants detected by RNA-seq

FN = Reference variants not detected by RNA-seq

The precision, which is a measure of true positives, was evaluated using GBS variant data as reference with the following equation:

Precision = 100* TP/(TP+FP)

where TP = GBS variants detected by RNA-seq

FP = Variants detected by RNA-seq but absent in GBS

The accuracy of genotype calls was evaluated by comparing the non-missing RNA-seq genotypes to the non-missing imputed BovineHD reference genotypes with a custom Perl script (Additional file 2) called using the above-mentioned bash pipeline.

## Statistical analysis

For all metrics (sensitivity, precision and the accuracy of genotype calls) the test for equality of variances indicated no significant difference in variances between the two variant calling methods for each minRD taken separately. Significant differences in central tendencies between the two variant calling methods were assessed using a Wilcoxon nonparametric test without matching the samples.

# References

1. Dobin A., Davis C.A., Schlesinger F., Drenkow J., Zaleski C., Jha S., et al. (2013) STAR: ultrafast universal RNA-seq aligner. Bioinformatics 29, 15-21.
2. McKenna A., Hanna M., Banks E., Sivachenko A., Cibulskis K., Kernytsky A., et al. (2010) The Genome Analysis Toolkit: a MapReduce framework for analyzing next-generation DNA sequencing data. Genome Res 20, 1297-303.
3. The Broad Institute (2017) GATK | Methods and Algorithms | Doc #7363 | Calling variants on cohorts of samples using the HaplotypeCaller in GVCF mode. URL https://software.broadinstitute.org/gatk/documentation/article.php?id=3893.
4. Brouard J.S., Boyle B., Ibeagha-Awemu E.M. & Bissonnette N. (2017) Low-depth genotyping-by-sequencing (GBS) in a bovine population: strategies to maximize the selection of high quality genotypes and the accuracy of imputation. BMC Genet 18, 32.
5. Sargolzaei M., Chesnais J.P. & Schenkel F.S. (2014) A new approach for efficient genotype imputation using information from relatives. BMC Genomics 15, 478.
6. Torkamaneh D., Laroche J., Bastien M., Abed A. & Belzile F. (2017) Fast-GBS: a new pipeline for the efficient and highly accurate calling of SNPs from genotyping-by-sequencing data. BMC Bioinformatics 18, 5.
