# Supplementary material for: The GATK joint genotyping workflow is appropriate for calling variants in RNA-seq experiments
Source: J Anim Sci Biotechnol. 2019 Jun 21;10:44. doi: 10.1186/s40104-019-0359-0 (PMC6587293; doi:10.1186/s40104-019-0359-0)
Supplement: Supplementary file 3 — Bioinformatics scripts used in this study. (DOCX 18 kb) [file 40104_2019_359_MOESM3_ESM.docx]

**evaluate_variant_calling_method.sh**

#!/bin/bash

set -e

set -u

set -o pipefail

# Usage ./evaluate_variant_calling_method.sh samples_list reference(gbs or hd) evaluated_method (joint-genotyping or per-sample) minRD

# Example ./evaluate_variant_calling_method.sh samples hd joint-genotyping 4

# some paths

GBS_PATH='../../../data/VCF/GBS/gbs.vcf.gz'

HD_PATH='../../../data/VCF/HD/hd.vcf.gz'

JGM_PATH='../../../data/VCF/joint-genotyping-method/jgm.vcf.gz'

PERSAMPLE_PATH='../../../data/VCF/per-sample-method/mm2.v6.vcf.gz'

SHARED_COVERED_SEQUENCES='../../../data/bedtools_genomeCov/shared_covered_sequences'

GENOMECOV_RNASEQ='../../../data/bedtools_genomeCov/genomeCov_RNASEQ'

# When GBS is used as reference

if [ $2 = "gbs" ]

then

echo "GBS variants are used as reference"

# Determination of the tested variant calling method

if [ $3 = "joint-genotyping" ]

then

echo "RNA method is joint-genotyping"

WORKINGDIR="../analysis/eval_variant_calling_method/GBS_DP$4_Joint-genotyping/"

else

if [ $3 = "per-sample" ]

then

echo "RNA method is Per-sample"

WORKINGDIR="../analysis/eval_variant_calling_method/GBS_DP$4_Per-sample/"

else

echo "specify either per-sample or joint-genotyping" && exit 1

fi

fi

# Creation of directories in /analysis

mkdir -p $WORKINGDIR

mkdir -p $WORKINGDIR/output

cp $1 $WORKINGDIR

cd $WORKINGDIR

touch ./output/log_GBS.csv

rm ./output/log_GBS.csv

# Write of the first line of output file

printf "sample,TP(detected RNASEQ variants presents in reference),\

TP+FN (all variants in reference),sensitivity, TP+FP \

(all variants detected in RNASEQ) \n">> output/log_GBS.csv

# Foreach sample

for i in $(cat $1); do

echo Processing $i

echo "$i.shared.covered.sequences.DP$4.txt"

# Check that the genomeCov file exist at the proper location

# When GBS is the reference, the file used lists regions covered by a specific minDP in GBS AND in RNASEQ

[ -f $SHARED_COVERED_SEQUENCES/"$i.shared.covered.sequences.DP$4.txt" ] && echo "shared genomeCov_ file exist" || (echo "shared genomeCov_ file does not exist. End of program!" && exit 1 )

echo $i | tr -d '\n' >>output/log_GBS.csv

# Preparation of GBS reference VCF file

bcftools view \

-s $i $GBS_PATH \

-R $SHARED_COVERED_SEQUENCES/$i.shared.covered.sequences.DP$4.txt > $i.ref.GBS.vcf

# Sorting

vcf-sort $i.ref.GBS.vcf > $i.ref.GBS.sorted.vcf

# Filtering to remove '0/0' and './.' genotypes

bcftools filter -e 'FORMAT/GT="0/0"' $i.ref.GBS.sorted.vcf > $i.ref.GBS.sorted.1.vcf

bcftools filter -e 'FORMAT/GT~"\."' $i.ref.GBS.sorted.1.vcf > $i.ref.GBS.filtered.2.vcf

# Clean-up step

rm $i.ref.GBS.vcf

rm $i.ref.GBS.sorted.1.vcf

# Preparation of RNASEQ detected variants (VCF file)

if [ "$3" = "joint-genotyping" ]

then

bcftools view \

-s $i $JGM_PATH \

-R $SHARED_COVERED_SEQUENCES/$i.shared.covered.sequences.DP$4.txt > $i.rna_gbs.vcf

else

if [ "$3" = "per-sample" ]

then

bcftools view \

-s $i $PERSAMPLE_PATH \

-R $SHARED_COVERED_SEQUENCES/$i.shared.covered.sequences.DP$4.txt > $i.rna_gbs.vcf

fi

fi

# Sorting

vcf-sort $i.rna_gbs.vcf > $i.rna_gbs.sorted.vcf

# Filtering to remove '0/0' and './.' genotypes

bcftools filter -e 'FORMAT/GT="0/0"' $i.rna_gbs.sorted.vcf > $i.rna_gbs.sorted.1.vcf

bcftools filter -e 'FORMAT/GT~"\."' $i.rna_gbs.sorted.1.vcf > $i.rna_gbs.filtered.2.vcf

# Clean-up step

rm $i.rna_gbs.vcf

rm $i.rna_gbs.sorted.1.vcf

# File preparation

bgzip -f $i.ref.GBS.filtered.2.vcf

tabix -p vcf $i.ref.GBS.filtered.2.vcf.gz

bgzip -f $i.rna_gbs.filtered.2.vcf

tabix -p vcf $i.rna_gbs.filtered.2.vcf.gz

# Intersection with BCFtools isec

bcftools isec -p $i/GBS_AS_REF $i.ref.GBS.filtered.2.vcf.gz $i.rna_gbs.filtered.2.vcf.gz

# Extraction of the number of variants in VCF files

# A - Detected variants that are part of reference variant (TP)

printf ",">>output/log_GBS.csv

bcftools view -H $i/GBS_AS_REF/0003.vcf | wc -l | tr -d '\n' >>output/log_GBS.csv

# B - All variants in the reference file (TP+FN)

gunzip $i.ref.GBS.filtered.2.vcf.gz

printf ",">>output/log_GBS.csv

bcftools view -H $i.ref.GBS.filtered.2.vcf | wc -l | tr -d '\n' >>output/log_GBS.csv

# C - All detected variants in RNASEQ (TP+FP)

gunzip $i.rna_gbs.filtered.2.vcf.gz

printf ",">>output/log_GBS.csv

bcftools view -H $i.rna_gbs.filtered.2.vcf | wc -l | tr -d '\n' >>output/log_GBS.csv

printf ",">>output/log_GBS.csv

# D - Evaluation of accuracy og genotype calls

../../../scripts/test_genotype_accuracy2.pl $i.ref.GBS.sorted.vcf $i.rna_gbs.sorted.vcf >>output/log_GBS.csv

printf "\n">>output/log_GBS.csv

done

# When HD is used as reference

else

if [ $2 = "hd" ]

then

echo "HD variants are used as reference"

# Determination of the tested variant calling method

if [ $3 = "joint-genotyping" ]

then

echo "RNA method is joint-genotyping"

WORKINGDIR="../analysis/eval_variant_calling_method/HD_DP$4_Joint-genotyping/"

else

if [ $3 = "per-sample" ]

then

echo "RNA method is Per-sample"

WORKINGDIR="../analysis/eval_variant_calling_method/HD_DP$4_Per-sample/"

else

echo "specify either per-sample or joint-genotyping" && exit 1

fi

fi

# Creation of directories in /analysis

mkdir -p $WORKINGDIR

mkdir -p $WORKINGDIR/output

cp $1 $WORKINGDIR

cd $WORKINGDIR

touch ./output/log_HD.csv

rm ./output/log_HD.csv

# Write of the first line of output file

printf "sample,TP(detected RNASEQ variants presents in reference),\

TP+FN (all variants in reference),sensibilite, TP+FP \

(all variants detected in RNASEQ) \n">> output/log_HD.csv

# Foreach sample

for i in $(cat $1); do

echo processing $i

# Check that the genomeCov file exist at the proper location

# When HD is the reference the file used lists regions covered by a specific minDP in RNASEQ file only

[ -f $GENOMECOV_RNASEQ/"$i.RNASEQ.genomeCov.txt.filtered.minDP.$4.txt" ] && echo "genomeCov_RNASEQ file exist" || (echo "genomeCov_RNASEQ does not exist. End of program!" && exit 1 )

echo $i | tr -d '\n' >>output/log_HD.csv

# Preparation of HD reference VCF file

bcftools view \

-s $i $HD_PATH \

-R $GENOMECOV_RNASEQ/"$i.RNASEQ.genomeCov.txt.filtered.minDP.$4.txt" > $i.ref.HD.vcf

# Sorting

vcf-sort $i.ref.HD.vcf > $i.ref.HD.sorted.vcf

# Filtering to remove '0/0' and './.' genotypes

bcftools filter -e 'FORMAT/GT="0/0"' $i.ref.HD.sorted.vcf > $i.ref.HD.sorted.1.vcf

bcftools filter -e 'FORMAT/GT~"\."' $i.ref.HD.sorted.1.vcf > $i.ref.HD.filtered.2.vcf

# Clean-up step

rm $i.ref.HD.vcf

rm $i.ref.HD.sorted.1.vcf

# Preparation of RNASEQ detected variants (VCF file)

if [ "$3" = "joint-genotyping" ]

then

bcftools view \

-s $i $JGM_PATH \

-R $i.ref.HD.sorted.vcf > $i.rna_HD.vcf

else

if [ "$3" = "per-sample" ]

then

bcftools view \

-s $i $PERSAMPLE_PATH \

-R $i.ref.HD.sorted.vcf > $i.rna_HD.vcf

fi

fi

# Sorting

vcf-sort $i.rna_HD.vcf > $i.rna_HD.sorted.vcf

# Filtering to remove '0/0' and './.' genotypes

bcftools filter -e 'FORMAT/GT="0/0"' $i.rna_HD.sorted.vcf > $i.rna_HD.sorted.1.vcf

bcftools filter -e 'FORMAT/GT~"\."' $i.rna_HD.sorted.1.vcf > $i.rna_HD.filtered.2.vcf

# Clean-up step

rm $i.rna_HD.vcf

rm $i.rna_HD.sorted.1.vcf

# File preparation

bgzip -f $i.ref.HD.filtered.2.vcf

tabix -p vcf $i.ref.HD.filtered.2.vcf.gz

bgzip -f $i.rna_HD.filtered.2.vcf

tabix -p vcf $i.rna_HD.filtered.2.vcf.gz

# Intersection with BCFtools isec

bcftools isec -p $i/HD_AS_REF $i.ref.HD.filtered.2.vcf.gz $i.rna_HD.filtered.2.vcf.gz

# Extraction of the number of variants in VCF files

# A - Detected variants that are part of reference variant (TP)

printf ",">>output/log_HD.csv

bcftools view -H $i/HD_AS_REF/0003.vcf | wc -l | tr -d '\n' >>output/log_HD.csv

# B - All variants in the reference file (TP+FN)

gunzip $i.ref.HD.filtered.2.vcf.gz

printf ",">>output/log_HD.csv

bcftools view -H $i.ref.HD.filtered.2.vcf | wc -l | tr -d '\n' >>output/log_HD.csv

# C - All detected variants in RNASEQ (TP+FP)

gunzip $i.rna_HD.filtered.2.vcf.gz

printf ",">>output/log_HD.csv

bcftools view -H $i.rna_HD.filtered.2.vcf | wc -l | tr -d '\n' >>output/log_HD.csv

printf ",">>output/log_HD.csv

# D - Evaluation of accuracy of genotype calls

../../../scripts/test_genotype_accuracy2.pl $i.ref.HD.sorted.vcf $i.rna_HD.sorted.vcf >>output/log_HD.csv

printf "\n">>output/log_HD.csv

done

fi

fi

**test_genotype_accuracy2.pl**

#!/usr/bin/perl

use strict;

use Cwd 'abs_path';

use warnings;

# author : Jean-Simon Brouard ; email:soda460@gmail.com

# usage : perl ./test_genotype_accuracy.pl investigated_VCF reference_VCF

# Description: This script evaluate the concordance of shared genotypes between

# those present in the investigated file and those present in the reference file

# Note : this script require that both files have the sames samples in the same order.

# If it is not the case, the % of accuracy will be lower than what is expected

unless (open(MYFILE1, "$ARGV[0]")) {

die ("Cannot open investigated vcf file!\n");

}

unless (open(MYFILE2, "$ARGV[1]")) {

die ("Cannot open reference vcf file!\n");

}

my $abs_path = abs_path($ARGV[0]);

my @file1 = <MYFILE1>;

my @file2 = <MYFILE2>;

chomp @file1;

chomp @file2;

# Declaration of variables

my %variant;

my %ref_variant;

my @array; # store the genotypes that are compared

my @ref_array; # store the reference genotypes

my $goodCalls = 0;

my $misCalls = 0;

my $missing_data_ref = 0;

my $missing_data = 0;

my @bad;

my $snps_tested = 0;

my @sample_names;

my $nb_samples;

# Data processing for the first file

foreach my $line (@file1) {

if ($line =~ /^chr/) {

$line =~ s/^chr//;

}

# Capture of sample name - case with only 1 sample

if ($line =~ /FORMAT\s+(\S+)$/) {

@sample_names = $1;

$nb_samples = 1;

}

# Capture of samples names

if ($line =~ /FORMAT\s+((\S+)\s+)+/) {

# Removal of FORMAT and all things before on that line

$line =~ s/(^.+FORMAT)(.+$)/$2/;

# Capture of samples names

@sample_names = split (/\s+/, $line);

shift @sample_names;

$nb_samples = @sample_names;

}

# Initialisation of variables

my $variant_name;

# Capture of variant name (key=CHROM:POSI:REF:ALT)

if ($line =~ /^(\S+)\s+(\d+)\s+(\S+)\s+([ATGCN]+)\s+([ATGCN]+)/) {

$variant_name="$1:$2:$4:$5";

$variant{$variant_name}{name} = $variant_name;

# Genotypes field of %variant is filled with genotypes

if ($line =~ /((\s+[.01][\/|][.01]\S*){$nb_samples})/) {

$variant{$variant_name}{genotypes} = "$1";

} else {

delete $variant{$variant_name};

next;

}

} else {

# print "ligne non capture";

next;

}

}

# Data processing for the second file, which is the reference file

foreach my $line2 (@file2) {

if ($line2 =~ /^#/) {

next;

}

if ($line2 =~ /^chr/) {

$line2 =~ s/^chr//;

}

# Initialisation of variables

my $ref_variant_name;

# Capture of variant name (key=CHROM:POSI:REF:ALT)

if ($line2 =~ /^(\S+)\s+(\d+)\s+(\S+)\s+([ATGCN]+)\s+([ATGCN]+)/) {

$ref_variant_name="$1:$2:$4:$5";

# Genotypes field of %variant is filled with genotypes

if ($line2 =~ /((\s+[.01][\/|][.01]\S*){$nb_samples})/) {

$ref_variant{$ref_variant_name}{genotypes} = "$1";

}

}

}

# Iterating over the elements of %variant (investigated file)

foreach my $clef (keys %variant)

{

# When a variant with the same key exists in the data

# structure associated to the second file (%ref_variant)

if (exists $ref_variant{$clef}{genotypes}) {

$snps_tested++;

# Genotypes field is split in individual genotypes

@ref_array = split (/\s+/, $ref_variant{$clef}{genotypes});

shift @ref_array; # remove the first array element, which is a blank

@array = split(/\s+/, $variant{$clef}{genotypes});

shift @array;

# Transformation of genotypes from 0/1 0|1 to 01

my $k = 0;

while ($k < @array) {

$array[$k] =~ s/([.01])[\/|]([.01])\S*/$1$2/;

$k++;

}

# Transformation of genotypes from 0/1 0|1 to 01

my $j = 0;

while ($j < @ref_array) {

$ref_array[$j] =~ s/([.01])[\/|]([.01])\S*/$1$2/;

$j++;

}

# Comparison of both arrays

my $i = 0;

while ($i < @array) {

# Case where the genotype from investigated file is unknown for that sample

if ($array[$i] eq '..') {

$missing_data++;

$i++;

next;

# Case where the genotype from investigated file is known for that sample

} else {

# When genotype is known in the reference file

if ($ref_array[$i] ne '..') {

# A - Case when the reference genotype is heterozygous

if ($ref_array[$i] eq '01' || $ref_array[$i] eq '10') {

# A1 - When both genotypes are concordant

if ($array[$i] eq '01' || $array[$i] eq '10') {

$goodCalls++;

$i++;

next;

# A2 - When genotypes are discordant

} else {

$misCalls++;

push (@bad, $array[$i], " vs ", $ref_array[$i], " \n");

$i++;

next;

}

}

# B - Case where the reference genotype is homozygous

if ($ref_array[$i] eq '00' || $ref_array[$i] eq '11' ) {

# B1 - When both genotypes are concordant

if ($array[$i] eq $ref_array[$i]) {

$goodCalls++;

# B2 - When genotypes are discordant

} else {

$misCalls++;

push (@bad, $array[$i], " vs ", $ref_array[$i], " \n");

}

}

# Case where ref array genotype eq '..', i.e is missing

} else {

$missing_data_ref++;

}

}

$i++;

}

}

}

my $accuracy = ($goodCalls/($goodCalls+$misCalls))*100;

my $arrondi = sprintf("%.1f", $accuracy);

my $missing = 0;

my $total = $goodCalls + $misCalls + $missing_data + $missing_data_ref;

$missing = $missing_data + $missing_data_ref;

my $percentage_missing = sprintf("%.1f", (($missing/$total)*100));

my $nb_tested = $goodCalls + $misCalls;

#print("--> The accuracy is ", $arrondi, "% (", $goodCalls, " identical Calls and ", $misCalls, " missCalls) ; ", $snps_tested, " variants tested \n");

print $arrondi;
